# Tight Bounds on the Rényi Entropy via Majorization with Applications to Guessing and Compression

**DOI:** 10.3390/e20120896

**Published:** 2018-11-22

**Authors:** Igal Sason

**Affiliations:** Department of Electrical Engineering, Technion—Israel Institute of Technology, Haifa 3200003, Israel; sason@ee.technion.ac.il; Tel.: +972-4-8294699

**Keywords:** Majorization, Rényi entropy, Rényi divergence, cumulant generating functions, guessing moments, lossless source coding, fixed-to-variable source codes, Huffman algorithm, Tunstall codes

## Abstract

This paper provides tight bounds on the Rényi entropy of a function of a discrete random variable with a finite number of possible values, where the considered function is not one to one. To that end, a tight lower bound on the Rényi entropy of a discrete random variable with a finite support is derived as a function of the size of the support, and the ratio of the maximal to minimal probability masses. This work was inspired by the recently published paper by Cicalese et al., which is focused on the Shannon entropy, and it strengthens and generalizes the results of that paper to Rényi entropies of arbitrary positive orders. In view of these generalized bounds and the works by Arikan and Campbell, non-asymptotic bounds are derived for guessing moments and lossless data compression of discrete memoryless sources.

## 1. Introduction

Majorization theory is a simple and productive concept in the theory of inequalities, which also unifies a variety of familiar bounds [1,2]. These mathematical tools find various applications in diverse fields (see, e.g., [3]) such as economics [2,4,5], combinatorial analysis [2,6], geometric inequalities [2], matrix theory [2,6,7,8], Shannon theory [5,9,10,11,12,13,14,15,16,17,18,19,20,21,22,23,24,25], and wireless communications [26,27,28,29,30,31,32,33].

This work, which relies on the majorization theory, has been greatly inspired by the recent insightful paper by Cicalese et al. [12] (the research work in the present paper has been initialized while the author handled [12] as an associate editor). The work in [12] provides tight bounds on the Shannon entropy of a function of a discrete random variable with a finite number of possible values, where the considered function is not one to one. For that purpose, and while being of interest by its own right (see [12], Section 6), a tight lower bound on the Shannon entropy of a discrete random variable with a finite support was derived in [12] as a function of the size of the support, and the ratio of the maximal to minimal probability masses. The present paper aims to extend the bounds in [12] to Rényi entropies of arbitrary positive orders (note that the Shannon entropy is equal to the Rényi entropy of order 1), and to study the information-theoretic applications of these (non-trivial) generalizations in the context of non-asymptotic analysis of guessing moments and lossless data compression.

The motivation for this work is rooted in the diverse information-theoretic applications of Rényi measures [34]. These include (but are not limited to) asymptotically tight bounds on guessing moments [35], information-theoretic applications such as guessing subject to distortion [36], joint source-channel coding and guessing with application to sequential decoding [37], guessing with a prior access to a malicious oracle [38], guessing while allowing the guesser to give up and declare an error [39], guessing in secrecy problems [40,41], guessing with limited memory [42], and guessing under source uncertainty [43]; encoding tasks [44,45]; Bayesian hypothesis testing [9,22,23], and composite hypothesis testing [46,47]; Rényi generalizations of the rejection sampling problem in [48], motivated by the communication complexity in distributed channel simulation, where these generalizations distinguish between causal and noncausal sampler scenarios [49]; Wyner’s common information in distributed source simulation under Rényi divergence measures [50]; various other source coding theorems [23,39,51,52,53,54,55,56,57,58], channel coding theorems [23,58,59,60,61,62,63,64], including coding theorems in quantum information theory [65,66,67].

The presentation in this paper is structured as follows: Section 2 provides notation and essential preliminaries for the analysis in this paper. Section 3 and Section 4 strengthen and generalize, in a non-trivial way, the bounds on the Shannon entropy in [12] to Rényi entropies of arbitrary positive orders (see Theorems 1 and 2). Section 5 relies on the generalized bound from Section 4 and the work by Arikan [35] to derive non-asymptotic bounds for guessing moments (see Theorem 3); Section 5 also relies on the generalized bound in Section 4 and the source coding theorem by Campbell [51] (see Theorem 4) for the derivation of non-asymptotic bounds for lossless compression of discrete memoryless sources (see Theorem 5).

## 2. Notation and Preliminaries

Let

- *P* be a probability mass function defined on a finite set X;
- $p_{\max}$ and $p_{\min}$ be, respectively, the maximal and minimal positive masses of *P*;
- $G_{P}\left(k\right)$ be the sum of the *k* largest masses of *P* for k∈{1,…,|X|} (note that $G_{P}\left(1\right)=p_{\max}$ and $G_{P}\left(|X|\right)=1$);
- $P_{n}$, for an integer n≥2, be the set of all probability mass functions defined on X with |X|=n; without any loss of generality, let X={1,…,n};
- $P_{n}\left(\rho \right)$, for ρ≥1 and an integer n≥2, be the subset of all probability measures $P\in P_{n}$ such that
  (1) $$\begin{array}{c} \frac{p_{\max}}{p_{\min}}\leq \rho . \end{array}$$

**Definition** **1** (Majorization)**.** *Consider discrete probability mass functions P and Q defined on the same (finite or countably infinite) set X. It is said that P is majorized by Q (or Q majorizes P), and it is denoted by P≺Q, if $G_{P}\left(k\right)\leq G_{Q}\left(k\right)$ for all k∈{1,…,|X|−1} (recall that $G_{P}\left(|X|\right)=G_{Q}\left(|X|\right)=1$). If P and Q are defined on finite sets of different cardinalities, then the probability mass function which is defined over the smaller set is first padded by zeros for making the cardinalities of these sets be equal.*

By Definition 1, a unit mass majorizes any other distribution; on the other hand, the equiprobable distribution on a finite set is majorized by any other distribution defined on the same set.

**Definition** **2** (Schur-convexity/concavity)**.** *A function $f:P_{n}\to R$ is said to be Schur-convex if for every $P,Q\in P_{n}$ such that P≺Q, we have f(P)≤f(Q). Likewise, f is said to be Schur-concave if −f is Schur-convex, i.e., $P,Q\in P_{n}$ and P≺Q imply that f(P)≥f(Q).*

**Definition** **3**  (Rényi entropy [34])**.** *Let X be a random variable taking values on a finite or countably infinite set X, and let $P_{X}$ be its probability mass function. The Rényi entropy of order α∈(0,1)∪(1,∞) is given by*

(2) $$\begin{array}{cc} H_{\alpha }\left(X\right) & =H_{\alpha }\left(P_{X}\right)=\frac{1}{1-\alpha }\;\log\left(\;\sum_{x\in X}P_{X}^{\alpha }\left(x\right)\right). \end{array}$$

*Unless explicitly stated, the logarithm base can be chosen by the reader, with *exp* indicating the inverse function of* log.

*By its continuous extension,*

(3) $$\begin{array}{c} H_{0}\left(X\right)=\log\;\left|\{x\in X:P_{X}\left(x\right)>0\}\right|, \end{array}$$

(4) $$\begin{array}{c} H_{1}\left(X\right)=H\left(X\right), \end{array}$$

(5) $$\begin{array}{c} H_{\infty }\left(X\right)=\log\frac{1}{p_{\max}} \end{array}$$

*where H(X) is the (Shannon) entropy of X.*

**Proposition** **1** (Schur-concavity of the Rényi entropy (Appendix F.3.a (p. 562) of [2]))**.** *The Rényi entropy of an arbitrary order α>0 is Schur-concave; in particular, for α=1, the Shannon entropy is Schur-concave.*

**Remark** **1.** *[17] (Theorem 2) strengthens Proposition 1, though it is not needed for our analysis.*

**Definition** **4**  (Rényi divergence [34])**.** *Let P and Q be probability mass functions defined on a finite or countably infinite set X. The Rényi divergence of order α∈[0,∞] is defined as follows:*

- *If α∈(0,1)∪(1,∞), then*
  (6) $$\begin{array}{c} D_{\alpha }\left(P\|Q\right)=\frac{1}{\alpha -1}\;\log\;\sum_{x\in X}P^{\alpha }\left(x\right)\;Q^{1-\alpha }\left(x\right). \end{array}$$
- *By the continuous extension of $D_{\alpha }\left(P\|Q\right)$,*
  (7) $$\begin{array}{cc} D_{0}\left(P\|Q\right) & =\max_{A:P(A)=1}\log\frac{1}{Q(A)}, \end{array}$$
  (8) $$\begin{array}{cc} D_{1}\left(P\|Q\right) & =D(P\|Q), \end{array}$$
  (9) $$\begin{array}{cc} D_{\infty }\left(P\|Q\right) & =\log\;\underset{x\in X}{\sup}\frac{P(x)}{Q(x)}, \end{array}$$
  *where D(P‖Q) in the right side of (8) is the relative entropy (a.k.a. Kullback-Leibler divergence).*

Throughout this paper, for a∈R, ⌈a⌉ denotes the ceiling of *a* (i.e., the smallest integer not smaller than the real number *a*), and ⌊a⌋ denotes the flooring of *a* (i.e., the greatest integer not greater than *a*).

## 3. A Tight Lower Bound on the Rényi Entropy

We provide in this section a tight lower bound on the Rényi entropy, of an arbitrary order α>0, when the probability mass function of the discrete random variable is defined on a finite set of cardinality *n*, and the ratio of the maximal to minimal probability masses is upper bounded by an arbitrary fixed value ρ∈[1,∞). In other words, we derive the largest possible gap between the order-α Rényi entropies of an equiprobable distribution and a non-equiprobable distribution (defined on a finite set of the same cardinality) with a given value for the ratio of the maximal to minimal probability masses. The basic tool used for the development of our result in this section relies on the majorization theory. Our result strengthens the result in [12] (Theorem 2) for the Shannon entropy, and it further provides a generalization for the Rényi entropy of an arbitrary order α>0 (recall that the Shannon entropy is equal to the Rényi entropy of order α=1, see (4)). Furthermore, the approach for proving the main result in this section differs significantly from the proof in [12] for the Shannon entropy. The main result in this section is a key result for all what follows in this paper.

The following lemma is a restatement of [12] (Lemma 6).

**Lemma** **1.** *Let $P\in P_{n}\left(\rho \right)$ with ρ≥1 and an integer n≥2, and assume without any loss of generality that the probability mass function P is defined on the set X={1,…,n}. Let $Q\in P_{n}$ be defined on X as follows:*

(10) $$Q\left(j\right)=\{\begin{array}{cc} \rho \;p_{\min}, & j\in \{1,\ldots ,i\},\\ 1-\left(n+i\rho -i-1\right)p_{\min}, & j=i+1,\\ p_{\min}, & j\in \{i+2,\ldots ,n\} \end{array}$$

*where*

(11) $$\begin{array}{c} i:=\left\lfloor \frac{1-np_{\min}}{\left(\rho -1\right)\;p_{\min}}\right\rfloor . \end{array}$$

*Then,*

*(1)*  *$Q\in P_{n}\left(\rho \right)$, and Q(1)≥Q(2)≥…≥Q(n)>0;*
*(2)*  *P≺Q.*

**Proof.**  See [12] (p. 2236) (top of the second column). ☐

**Lemma** **2.** *Let ρ>1, α>0, and n≥2 be an integer. For*

(12) $$\begin{array}{c} \beta \in \left[\frac{1}{1+(n-1)\rho },\;\frac{1}{n}\right]:=\Gamma_{\rho }^{\left(n\right)}, \end{array}$$

*let $Q_{\beta }\in P_{n}\left(\rho \right)$ be defined on X={1,…,n} as follows:*

(13) $$Q_{\beta }\left(j\right)=\{\begin{array}{cc} \rho \beta , & \;j\in \{1,\ldots ,i_{\beta }\},\\ 1-(n+i_{\beta }\;\rho -i_{\beta }-1)\beta , & \;j=i_{\beta }+1,\\ \beta , & \;j\in \{i_{\beta }+2,\ldots ,n\} \end{array}$$

*where*

(14) $$\begin{array}{c} i_{\beta }:=\left\lfloor \frac{1-n\beta }{(\rho -1)\beta }\right\rfloor . \end{array}$$

*Then, for every α>0,*

(15) $$\begin{array}{c} \min_{P\in P_{n}\left(\rho \right)}H_{\alpha }\left(P\right)=\min_{\beta \in \Gamma_{\rho }^{\left(n\right)}}H_{\alpha }\left(Q_{\beta }\right). \end{array}$$

**Proof.**  See Appendix A. ☐

**Lemma** **3.** *For ρ>1 and α>0, let*

(16) $$\begin{array}{c} c_{\alpha }^{\left(n\right)}\left(\rho \right):=\log n-\min_{P\in P_{n}\left(\rho \right)}H_{\alpha }\left(P\right),\;n=2,3,\ldots \end{array}$$

*with $c_{\alpha }^{\left(1\right)}\left(\rho \right):=0$. Then, for every n∈N,*

(17) $$\begin{array}{c} 0\leq c_{\alpha }^{\left(n\right)}\left(\rho \right)\leq \log\rho , \end{array}$$

(18) $$\begin{array}{c} c_{\alpha }^{\left(n\right)}\left(\rho \right)\leq c_{\alpha }^{\left(2n\right)}\left(\rho \right), \end{array}$$

*and $c_{\alpha }^{\left(n\right)}\left(\rho \right)$ is monotonically increasing in α∈[0,∞].*

**Proof.**  See Appendix B. ☐

**Lemma** **4.** *For α>0 and ρ>1, the limit*

(19) $$\begin{array}{cc} c_{\alpha }^{\left(\infty \right)}\left(\rho \right) & :=\lim_{n\to \infty }c_{\alpha }^{\left(n\right)}\left(\rho \right) \end{array}$$

*exists, having the following properties:*

*(a)*  *If α∈(0,1)∪(1,∞), then*
  (20) $$\begin{array}{c} c_{\alpha }^{\left(\infty \right)}\left(\rho \right)=\frac{1}{\alpha -1}\;\log\left(1+\frac{1+\alpha \;\left(\rho -1\right)-\rho^{\alpha }}{\left(1-\alpha )(\rho -1\right)}\right)-\frac{\alpha }{\alpha -1}\;\log\left(1+\frac{1+\alpha \;\left(\rho -1\right)-\rho^{\alpha }}{\left(1-\alpha \right)\left(\rho^{\alpha }-1\right)}\right), \end{array}$$
  *and*
  (21) $$\begin{array}{c} \lim_{\alpha \to \infty }c_{\alpha }^{\left(\infty \right)}\left(\rho \right)=\log\rho . \end{array}$$
*(b)*  *If α=1, then*
  (22) $$\begin{array}{c} c_{1}^{\left(\infty \right)}\left(\rho \right)=\lim_{\alpha \to 1}c_{\alpha }^{\left(\infty \right)}\left(\rho \right)=\frac{\rho \log\rho }{\rho -1}-\log\left(\frac{e\rho \log_{e}\rho }{\rho -1}\right). \end{array}$$
*(c)*  *For all α>0,*
  (23) $$\begin{array}{c} \lim_{\rho ↓1}c_{\alpha }^{\left(\infty \right)}\left(\rho \right)=0. \end{array}$$

- *For every n∈N, α>0 and ρ≥1,*
  (24) $$\begin{array}{c} 0\leq c_{\alpha }^{\left(n\right)}\left(\rho \right)\leq c_{\alpha }^{\left(2n\right)}\left(\rho \right)\leq c_{\alpha }^{\left(\infty \right)}\left(\rho \right)\leq \log\rho . \end{array}$$

**Proof.**  See Appendix C. ☐

In view of Lemmata 1–4, we obtain the following main result in this section:

**Theorem** **1.** *Let α>0, ρ>1, n≥2, and let $c_{\alpha }^{\left(n\right)}\left(\rho \right)$ in (16) designate the maximal gap between the order-α Rényi entropies of equiprobable and arbitrary distributions in $P_{n}\left(\rho \right)$. Then,*

*(a)*  *The non-negative sequence ${\left\{c_{\alpha }^{\left(n\right)}\left(\rho \right)\right\}}_{n=2}^{\infty }$ can be calculated by the real-valued single-parameter optimization in the right side of (15).*
*(b)*  *The asymptotic limit as n→∞, denoted by $c_{\alpha }^{\left(\infty \right)}\left(\rho \right)$, admits the closed-form expressions in (20) and (22), and it satisfies the properties in (21), (23) and (24).*

**Remark** **2.** *Setting α=2 in Theorem 1 gives that, for all $P\in P_{n}\left(\rho \right)$ (with ρ>1, and an integer n≥2),*

(25) $$\begin{array}{cc} H_{2}\left(P\right) & \geq \log n-c_{2}^{\left(n\right)}\left(\rho \right) \end{array}$$

(26) $$\begin{array}{cc} & \geq \log n-c_{2}^{\left(\infty \right)}\left(\rho \right) \end{array}$$

(27) $$\begin{array}{cc} & =\log\frac{4\rho n}{{\left(1+\rho \right)}^{2}} \end{array}$$

*where (25)–(27) hold, respectively, due to (16), (24) and (20). This strengthens the result in [68] (Proposition 2) which gives the same lower bound as in the right side of (27) for H(P) rather than for $H_{2}\left(P\right)$ (recall that $H\left(P\right)\geq H_{2}\left(P\right)$).*

**Figure 1:**
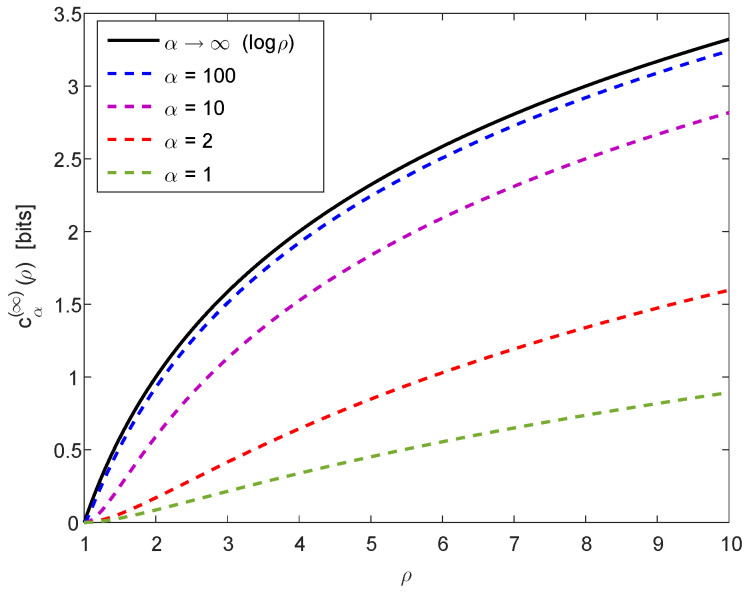
A plot of $c_{\alpha }^{\left(\infty \right)}\left(\rho \right)$ in (20) and (22) (log is on base 2) as a function of ρ, confirming numerically the properties in (21) and (23).

**Figure 2:**
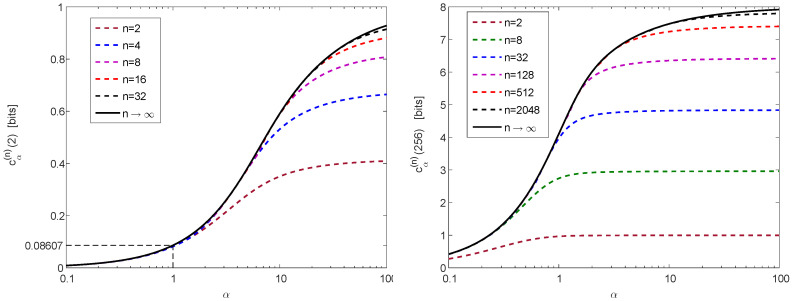
Plots of $c_{\alpha }^{\left(n\right)}\left(\rho \right)$ in (16) (log is on base 2) as a function of α>0, for ρ=2 (left plot) and ρ=256 (right plot), with several values of n≥2.

For a numerical illustration of Theorem 1, Figure 1 provides a plot of $c_{\alpha }^{\left(\infty \right)}\left(\rho \right)$ in (20) and (22) as a function of ρ≥1, confirming numerically the properties in (21) and (23). Furthermore, Figure 2 provides plots of $c_{\alpha }^{\left(n\right)}\left(\rho \right)$ in (16) as a function of α>0, for ρ=2 (left plot) and ρ=256 (right plot), with several values of n≥2; the calculation of the curves in these plots relies on (15), (20) and (22), and they illustrate the monotonicity and boundedness properties in (24).

**Remark** **3.** *Theorem 1 strengthens the result in [12] (Theorem 2) for the Shannon entropy (i.e., for α=1), in addition to its generalization to Rényi entropies of arbitrary orders α>0. This is because our lower bound on the Shannon entropy is given by*

(28) $$\begin{array}{c} H\left(P\right)\geq \log n-c_{1}^{\left(n\right)}\left(\rho \right),\;\forall \;P\in P_{n}\left(\rho \right), \end{array}$$

*whereas the looser bound in [12] is given by (see [12] ((7)) and (22) here)*

(29) $$\begin{array}{c} H\left(P\right)\geq \log n-c_{1}^{\left(\infty \right)}\left(\rho \right),\;\forall \;P\in P_{n}\left(\rho \right), \end{array}$$

**Figure 3:**
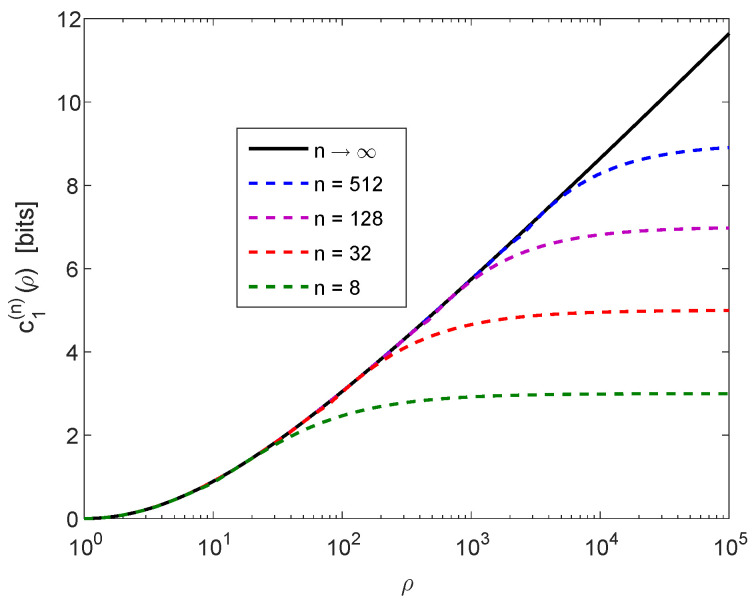
A plot of $c_{1}^{\left(\infty \right)}\left(\rho \right)$ in (22) versus $c_{1}^{\left(n\right)}\left(\rho \right)$ for finite n (n=512,128,32, and 8) as a function of *ρ*.

*and we recall that $0\leq c_{1}^{\left(n\right)}\left(\rho \right)\leq c_{1}^{\left(\infty \right)}\left(\rho \right)$ (see (24)). Figure 3 shows the improvement in the new lower bound (28) over (29) by comparing $c_{1}^{\left(\infty \right)}\left(\rho \right)$ versus $c_{1}^{\left(n\right)}\left(\rho \right)$ for $\rho \in [1,10^{5}]$ and with several values of n. It is reflected from Figure 3 that there is a very marginal improvement in the lower bound on the Shannon entropy (28) over the bound in (29) if ρ≤30 (even for small values of n), whereas there is a significant improvement over the bound in (29) for large values of ρ; by increasing the value of n, also the value of ρ needs to be increased for observing an improvement of the lower bound in (28) over (29) (see Figure 3).*

*An improvement of the bound in (28) over (29) leads to a tightening of the upper bound in [12] (Theorem 4) on the compression rate of Tunstall codes for discrete memoryless sources, which further tightens the bound by Jelinek and Schneider in [69] (Equation (9)). More explicitly, in view of [12] (Section 6), an improved upper bound on the compression rate of these variable-to-fixed lossless source codes is obtained by combining [12] (Equations (36) and (38)) with a tightened lower bound on the entropy H(W) of the leaves of the tree graph for Tunstall codes. From (28), the latter lower bound is given by $H\left(W\right)\geq \log_{2}n-c_{1}^{\left(n\right)}\left(\rho \right)$ where $c_{1}^{\left(n\right)}\left(\rho \right)$ is expressed in bits, $\rho :=\frac{1}{p_{\min}}$ is the reciprocal of the minimal positive probability of the source symbols, and n is the number of codewords (so, all codewords are of length $\left\lceil \log_{2}n\right\rceil$ bits). This yields a reduction in the upper bound on the non-asymptotic compression rate R of Tunstall codes from $\frac{\left\lceil \log_{2}n\right\rceil \;H\left(X\right)}{\log_{2}n-c_{1}^{\left(\infty \right)}\left(\rho \right)}$ (see [12] (Equation (40)) and (22)) to $\frac{\left\lceil \log_{2}n\right\rceil \;H\left(X\right)}{\log_{2}n-c_{1}^{\left(n\right)}\left(\rho \right)}$ bits per source symbol where H(X) denotes the source entropy (converging, in view of (17), to H(X) as we let n→∞).*

**Remark** **4.** *Equality (15) with the minimizing probability mass function of the form (13) holds, in general, by replacing the Rényi entropy with an arbitrary Schur-concave function (as it can be easily verified from the proof of Lemma 2 in Appendix A). However, the analysis leading to Lemmata 3–4 and Theorem 1 applies particularly to the Rényi entropy.*

## 4. Bounds on the Rényi Entropy of a Function of a Discrete Random Variable

This section relies on Theorem 1 and majorization for extending [12] (Theorem 1), which applies to the Shannon entropy, to Rényi entropies of any positive order. More explicitly, let α∈(0,∞) and

- X and Y be finite sets of cardinalities |X|=n and |Y|=m with n>m≥2; without any loss of generality, let X={1,…,n} and Y={1,…,m};
- *X* be a random variable taking values on X with a probability mass function $P_{X}\in P_{n}$;
- $F_{n,m}$ be the set of deterministic functions f:X→Y; note that $f\in F_{n,m}$ is not one to one since m<n.

The main result in this section sharpens the inequality $H_{\alpha }\left(f(X)\right)\leq H_{\alpha }\left(X\right)$, for every deterministic function $f\in F_{n,m}$ with n>m≥2 and α>0, by obtaining non-trivial upper and lower bounds on $\max_{f\in F_{n,m}}H_{\alpha }\left(f(X)\right)$. The calculation of the exact value of $\min_{f\in F_{n,m}}H_{\alpha }\left(f(X)\right)$ is much easier, and it is expressed in closed form by capitalizing on the Schur-concavity of the Rényi entropy.

The following main result extends [12] (Theorem 1) to Rényi entropies of arbitrary positive orders.

**Theorem** **2.** *Let X∈{1,…,n} be a random variable which satisfies $P_{X}\left(1\right)\geq P_{X}\left(2\right)\geq \ldots \geq P_{X}\left(n\right)$.*

*(a)*  *For m∈{2,…,n−1}, if $P_{X}\left(1\right)<\frac{1}{m}$, let ${\tilde{X}}_{m}$ be the equiprobable random variable on {1,…,m}; otherwise, if $P_{X}\left(1\right)\geq \frac{1}{m}$, let ${\tilde{X}}_{m}\in \left\{1,\ldots ,m\right\}$ be a random variable with the probability mass function*
  (30) $$P_{{\tilde{X}}_{m}}\left(i\right)=\{\begin{array}{cc} P_{X}\left(i\right), & i\in \{1,\ldots ,n^{*}\},\\ \frac{1}{m-n^{*}}\sum_{j=n^{*}+1}^{n}P_{X}\left(j\right), & i\in \{n^{*}+1,\ldots ,m\}, \end{array}$$
  *where $n^{*}$ is the maximal integer i∈{1,…,m−1} such that*
  (31) $$\begin{array}{c} P_{X}\left(i\right)\geq \frac{1}{m-i}\sum_{j=i+1}^{n}P_{X}\left(j\right). \end{array}$$
*Then, for every α>0,*
  (32) $$\begin{array}{c} \max_{f\in F_{n,m}}H_{\alpha }\left(f(X)\right)\in \left[H_{\alpha }\left({\tilde{X}}_{m}\right)-v\left(\alpha \right),\;H_{\alpha }\left({\tilde{X}}_{m}\right)\right], \end{array}$$
  *where*
  (33) $$v\left(\alpha \right):=c_{\alpha }^{\left(\infty \right)}\left(2\right)=\{\begin{array}{cc} \log\left(\frac{\alpha -1}{2^{\alpha }-2}\right)-\frac{\alpha }{\alpha -1}\;\log\left(\frac{\alpha }{2^{\alpha }-1}\right), & \alpha \neq 1,\\ \log\left(\frac{2}{e\;\ln2}\right)\approx 0.08607\;bits, & \alpha =1. \end{array}$$
*(b)*  *There exists an explicit construction of a deterministic function $f^{*}\in F_{n,m}$ such that*
  (34) $$\begin{array}{c} H_{\alpha }\left(f^{*}\left(X\right)\right)\in \left[H_{\alpha }\left({\tilde{X}}_{m}\right)-v\left(\alpha \right),\;H_{\alpha }\left({\tilde{X}}_{m}\right)\right] \end{array}$$
  *where $f^{*}$ is independent of α, and it is obtained by using Huffman coding (as in [12] for α=1).*
*(c)*  *Let ${\tilde{Y}}_{m}\in \left\{1,\ldots ,m\right\}$ be a random variable with the probability mass function*
  (35) $$P_{{\tilde{Y}}_{m}}\left(i\right)=\{\begin{array}{cc} \sum_{k=1}^{n-m+1}P_{X}\left(k\right), & i=1,\\ P_{X}\left(n-m+i\right), & i\in \{2,\ldots ,m\}. \end{array}$$
*Then, for every α>0,*
  (36) $$\begin{array}{c} \min_{f\in F_{n,m}}H_{\alpha }\left(f(X)\right)=H_{\alpha }\left({\tilde{Y}}_{m}\right). \end{array}$$

**Remark** **5.** *Setting α=1 specializes Theorem 2 to [12] (Theorem 1) (regarding the Shannon entropy). This point is further elaborated in Remark 8, after the proof of Theorem 2.*

**Remark** **6.** *Similarly to [12] (Lemma 1), an exact solution of the maximization problem in the left side of (32) is strongly NP-hard [70]; this means that, unless P=NP, there is no polynomial time algorithm which, for an arbitrarily small ε>0, computes an admissible deterministic function $f_{\varepsilon }\in F_{n,m}$ such that*

(37) $$\begin{array}{c} H_{\alpha }\left(f_{\varepsilon }\left(X\right)\right)\geq \left(1-\varepsilon \right)\max_{f\in F_{n,m}}H_{\alpha }\left(f(X)\right). \end{array}$$

*This motivates the derivation of the bounds in (32), and the simple construction of a deterministic function $f^{*}\in F_{n,m}$ achieving (34).*

A proof of Theorem 2 relies on the following lemmata.

**Lemma** **5.** *Let X∈{1,…,n}, m<n and α>0. Then,*

(38) $$\begin{array}{c} \max_{Q\in P_{m}:\;P_{X}≺Q}\;H_{\alpha }\left(Q\right)=H_{\alpha }\left({\tilde{X}}_{m}\right) \end{array}$$

*where the probability mass function of ${\tilde{X}}_{m}$ is given in (30).*

**Proof.**  Since $P_{X}≺P_{{\tilde{X}}_{m}}$ (see [12] (Lemma 2)) with $P_{{\tilde{X}}_{m}}\in P_{m}$, and $P_{{\tilde{X}}_{m}}≺Q$ for all $Q\in P_{m}$ such that $P_{X}≺Q$ (see [12] (Lemma 4)), the result follows from the Schur-concavity of the Rényi entropy. ☐

**Lemma** **6.** *Let X∈{1,…,n}, α>0, and $f\in F_{n,m}$ with m<n. Then,*

(39) $$\begin{array}{c} H_{\alpha }\left(f(X)\right)\leq H_{\alpha }\left({\tilde{X}}_{m}\right). \end{array}$$

**Proof.**  Since *f* is a deterministic function in $F_{n,m}$ with m<n, the probability mass function of f(X) is an element in $P_{m}$ which majorizes $P_{X}$ (see [12] (Lemma 3)). Inequality (39) then follows from Lemma 5. ☐

We are now ready to prove Theorem 2.

**Proof.**  In view of (39),

(40) $$\begin{array}{c} \max_{f\in F_{n,m}}H_{\alpha }\left(f(X)\right)\leq H_{\alpha }\left({\tilde{X}}_{m}\right). \end{array}$$

We next construct a function $f^{*}\in F_{n,m}$ such that, for all α>0,

(41) $$\begin{array}{cc} H_{\alpha }\left(f^{*}\left(X\right)\right) & \geq \max_{Q\in P_{m}:\;P_{X}≺Q}\;H_{\alpha }\left(Q\right)-v\left(\alpha \right) \end{array}$$

(42) $$\begin{array}{c} \geq \max_{f\in F_{n,m}}H_{\alpha }\left(f(X)\right)-v\left(\alpha \right) \end{array}$$

where the function v:(0,∞)→(0,∞) in the right side of (41) is given in (33), and (42) holds due to (38) and (40). The function $f^{*}$ in our proof coincides with the construction in [12], and it is, therefore, independent of α.

We first review and follow the concept of the proof of [12] (Lemma 5), and we then deviate from the analysis there for proving our result. The idea behind the proof of [12] (Lemma 5) relies on the following algorithm:

(1) Start from the probability mass function $P_{X}\in P_{n}$ with $P_{X}\left(1\right)\geq \ldots \geq P_{X}\left(n\right)$;
(2) Merge successively pairs of probability masses by applying the Huffman algorithm;
(3) Stop the merging process in Step 2 when a probability mass function $Q\in P_{m}$ is obtained (with Q(1)≥…≥Q(m));
(4) Construct the deterministic function $f^{*}\in F_{n,m}$ by setting $f^{*}\left(k\right)=j\in \left\{1,\ldots ,m\right\}$ for all probability masses $P_{X}\left(k\right)$, with k∈{1,…,n}, being merged in Steps 2–3 into the node of Q(j).

Let i∈{0,…,m−1} be the largest index such that $P_{X}\left(1\right)=Q\left(1\right),\ldots ,P_{X}\left(i\right)=Q\left(i\right)$ (note that i=0 corresponds to the case where each node Q(j), with j∈{1,…,m}, is constructed by merging at least two masses of the probability mass function $P_{X}$). Then, according to [12] (p. 2225),

(43) $$\begin{array}{c} Q(i+1)\leq 2\;Q(m). \end{array}$$

Let

(44) $$\begin{array}{c} S:=\sum_{j=i+1}^{m}Q\left(j\right) \end{array}$$

be the sum of the m−i smallest masses of the probability mass function *Q*. In view of (43), the vector

(45) $$\begin{array}{c} \bar{Q}:=\left(\frac{Q(i+1)}{S},\ldots ,\frac{Q(m)}{S}\right) \end{array}$$

represents a probability mass function where the ratio of its maximal to minimal masses is upper bounded by 2.

At this point, our analysis deviates from [12] (p. 2225). Applying Theorem 1 to $\bar{Q}$ with ρ=2 gives

(46) $$\begin{array}{c} H_{\alpha }\left(\bar{Q}\right)\geq \log\left(m-i\right)-c_{\alpha }^{\left(\infty \right)}\left(2\right) \end{array}$$

with

(47) $$\begin{array}{cc} c_{\alpha }^{\left(\infty \right)}\left(2\right) & =\frac{1}{\alpha -1}\log\left(1+\frac{1+\alpha -2^{\alpha }}{1-\alpha }\right)-\frac{\alpha }{\alpha -1}\log\left(1+\frac{1+\alpha -2^{\alpha }}{\left(1-\alpha \right)\left(2^{\alpha }-1\right)}\right) \end{array}$$

(48) $$\begin{array}{c} =\log\left(\frac{\alpha -1}{2^{\alpha }-2}\right)-\frac{\alpha }{\alpha -1}\log\left(\frac{\alpha }{2^{\alpha }-1}\right) \end{array}$$

(49) $$\begin{array}{c} =v(\alpha ) \end{array}$$

where (47) follows from (20); (48) is straightforward algebra, and (49) is the definition in (33).

For α∈(0,1)∪(1,∞), we get

(50) $$\begin{array}{cc} H_{\alpha }\left(Q\right) & =\frac{1}{1-\alpha }\;\log\left(\sum_{j=1}^{m}Q^{\alpha }\left(j\right)\right) \end{array}$$

(51) $$\begin{array}{cc} & =\frac{1}{1-\alpha }\;\log\left(\sum_{j=1}^{i}Q^{\alpha }\left(j\right)+\sum_{j=i+1}^{m}Q^{\alpha }\left(j\right)\right) \end{array}$$

(52) $$\begin{array}{cc} & =\frac{1}{1-\alpha }\;\log\left(\sum_{j=1}^{i}Q^{\alpha }\left(j\right)+S^{\alpha }\;\exp\left(\left(1-\alpha \right)H_{\alpha }\left(\bar{Q}\right)\right)\right) \end{array}$$

(53) $$\begin{array}{cc} & \geq \frac{1}{1-\alpha }\;\log\left(\sum_{j=1}^{i}Q^{\alpha }\left(j\right)+S^{\alpha }\;\exp\left(\left(1-\alpha \right)\left(\log(m-i)-v(\alpha )\right)\right)\right) \end{array}$$

(54) $$\begin{array}{cc} & =\frac{1}{1-\alpha }\;\log\left(\sum_{j=1}^{i}Q^{\alpha }\left(j\right)+S^{\alpha }\;{\left(m-i\right)}^{1-\alpha }\exp\left((\alpha -1)\;v(\alpha )\right)\right) \end{array}$$

where (51) holds since i∈{0,…,m−1}; (52) follows from (2) and (45); (53) holds by (46)–(49).

In view of (44), let $Q^{*}\in P_{m}$ be the probability mass function which is given by

(55) $$Q^{*}\left(j\right)=\{\begin{array}{cc} Q(j), & j=1,\ldots ,i\\ \frac{S}{m-i}, & j=i+1,\ldots ,m. \end{array}$$

From (50)–(55), we get

(56) $$\begin{array}{cc} H_{\alpha }\left(Q\right) & \geq \frac{1}{1-\alpha }\;\log\left(\sum_{j=1}^{i}{\left(Q^{*}\left(j\right)\right)}^{\alpha }+\sum_{j=i+1}^{m}{\left(Q^{*}\left(j\right)\right)}^{\alpha }\;\exp\left((\alpha -1)\;v(\alpha )\right)\right) \end{array}$$

(57) $$\begin{array}{cc} & =\frac{1}{1-\alpha }\;\log\left(\sum_{j=1}^{m}{\left(Q^{*}\left(j\right)\right)}^{\alpha }+\sum_{j=i+1}^{m}{\left(Q^{*}\left(j\right)\right)}^{\alpha }\;\left(\exp\left((\alpha -1)\;v(\alpha )\right)-1\right)\right) \end{array}$$

(58) $$\begin{array}{cc} & =H_{\alpha }\left(Q^{*}\right)+\frac{1}{1-\alpha }\;\log\left(1+T\;\left(\exp\left((\alpha -1)\;v(\alpha )\right)-1\right)\right) \end{array}$$

with

(59) $$\begin{array}{c} T:=\frac{\sum_{j=i+1}^{m}{\left(Q^{*}\left(j\right)\right)}^{\alpha }}{\sum_{j=1}^{m}{\left(Q^{*}\left(j\right)\right)}^{\alpha }}\in \left[0,1\right]. \end{array}$$

Since T∈[0,1] and v(α)>0 for α>0, it can be verified from (56)–(58) that for α∈(0,1)∪(1,∞)

(60) $$\begin{array}{c} H_{\alpha }\left(Q\right)\geq H_{\alpha }\left(Q^{*}\right)-v\left(\alpha \right). \end{array}$$

The validity of (60) is extended to α=1 by taking the limit α→1 on both sides of this inequality, and due to the continuity of v(·) in (33) at α=1. Applying the majorization result $Q^{*}≺P_{{\tilde{X}}_{m}}$ in [12] ((31)), it follows from (60) and the Schur-concavity of the Rényi entropy that, for all α>0,

(61) $$\begin{array}{c} H_{\alpha }\left(Q\right)\geq H_{\alpha }\left(Q^{*}\right)-v\left(\alpha \right)\geq H_{\alpha }\left({\tilde{X}}_{m}\right)-v\left(\alpha \right), \end{array}$$

which together with (40), prove Items a) and b) of Theorem 2 (note that, in view of the construction of the deterministic function $f^{*}\in F_{n,m}$ in Step 4 of the above algorithm, we get $H_{\alpha }\left(f^{*}\left(X\right)\right)=H_{\alpha }\left(Q\right)$).

We next prove Item c). Equality (36) is due to the Schur-concavity of the Rényi entropy, and since we have

- f(X) is an aggregation of *X*, i.e., the probability mass function $Q\in P_{m}$ of f(X) satisfies $Q\left(j\right)=\sum_{i\in I_{j}}P_{X}\left(i\right)$ (1≤j≤m) where $I_{1},\ldots ,I_{m}$ partition {1,…,n} into *m* disjoint subsets as follows:
  (62) $$\begin{array}{c} I_{j}:=\left\{i\in \left\{1,\ldots ,n\right\}:f\left(i\right)=j\right\},\;j=1,\ldots ,m; \end{array}$$
- By the assumption $P_{X}\left(1\right)\geq P_{X}\left(2\right)\geq \ldots \geq P_{X}\left(n\right)$, it follows that $Q≺P_{{\tilde{Y}}_{m}}$ for every such $Q\in P_{m}$;
- From (35), ${\tilde{Y}}_{m}=\tilde{f}\left(X\right)$ where the function $\tilde{f}\in F_{n,m}$ is given by $\tilde{f}\left(k\right):=1$ for all k∈{1,…,n−m+1}, and $\tilde{f}\left(n-m+i\right):=i$ for all i∈{2,…,m}. Hence, $P_{{\tilde{Y}}_{m}}$ is an element in the set of the probability mass functions of f(X) with $f\in F_{n,m}$ which majorizes every other element from this set.

 ☐

**Remark** **7.** *The solid line in the left plot of Figure 2 depicts $v\left(\alpha \right):=c_{\alpha }^{\left(\infty \right)}\left(2\right)$ in (33) for α>0. In view of Lemma 4, and by the definition in (33), the function v:(0,∞)→(0,∞) is indeed monotonically increasing and continuous.*

**Remark** **8.** *Inequality (43) leads to the application of Theorem 1 with ρ=2 (see (46)). In the derivation of Theorem 2, we refer to $v\left(\alpha \right):=c_{\alpha }^{\left(\infty \right)}\left(2\right)$ (see (47)–(49)) rather than referring to $c_{\alpha }^{\left(n\right)}\left(2\right)$ (although, from (24), we have $0\leq c_{\alpha }^{\left(n\right)}\left(2\right)\leq v\left(\alpha \right)$ for all α>0). We do so since, for n≥16, the difference between the curves of $c_{\alpha }^{\left(n\right)}\left(2\right)$ (as a function of α>0) and the curve of $c_{\alpha }^{\left(\infty \right)}\left(2\right)$ is marginal (see the dashed and solid lines in the left plot of Figure 2), and also because the function v in (33) is expressed in a closed form whereas $c_{\alpha }^{\left(n\right)}\left(2\right)$ is subject to numerical optimization for finite n (see (15) and (16)). For this reason, Theorem 2 coincides with the result in [12] (Theorem 1) for the Shannon entropy (i.e., for α=1) while providing a generalization of the latter result for Rényi entropies of arbitrary positive orders α. Theorem 1, however, both strengthens the bounds in [12] (Theorem 2) for the Shannon entropy with finite cardinality n (see Remark 3), and it also generalizes these bounds to Rényi entropies of all positive orders.*

**Remark** **9.** *The minimizing probability mass function in (35) to the optimization problem (36), and the maximizing probability mass function in (30) to the optimization problem (38) are in general valid when the Rényi entropy of a positive order is replaced by an arbitrary Schur-concave function. However, the main results in (32)–(34) hold particularly for the Rényi entropy.*

**Remark** **10.** *Theorem 2 makes use of the random variables denoted by ${\tilde{X}}_{m}$ and ${\tilde{Y}}_{m}$, rather than (more simply) $X_{m}$ and $Y_{m}$ respectively, because Section 5 considers i.i.d. samples ${\left\{X_{i}\right\}}_{i=1}^{k}$ and ${\left\{Y_{i}\right\}}_{i=1}^{k}$ with $X_{i}∼P_{X}$ and $Y_{i}∼P_{Y}$; note, however, that the probability mass functions of ${\tilde{X}}_{m}$ and ${\tilde{Y}}_{m}$ are different from $P_{X}$ and $P_{Y}$, respectively, and for that reason we make use of tilted symbols in the left sides of (30) and (35).*

## 5. Information-Theoretic Applications: Non-Asymptotic Bounds for Lossless Compression and Guessing

Theorem 2 is applied in this section to derive non-asymptotic bounds for lossless compression of discrete memoryless sources and guessing moments. Each of the two subsections starts with a short background for making the presentation self-contained.

### 5.1. Guessing

#### 5.1.1. Background

The problem of guessing discrete random variables has various theoretical and operational aspects in information theory (see [35,36,37,38,40,41,43,56,71,72,73,74,75,76,77,78,79,80,81]). The central object of interest is the distribution of the number of guesses required to identify a realization of a random variable *X*, taking values on a finite or countably infinite set X={1,…,|X|}, by successively asking questions of the form “Is *X* equal to *x*?” until the value of *X* is guessed correctly. A guessing function is a one-to-one function g:X→X, which can be viewed as a permutation of the elements of X in the order in which they are guessed. The required number of guesses is therefore equal to g(x) when X=x with x∈X.

Lower and upper bounds on the minimal expected number of required guesses for correctly identifying the realization of *X*, expressed as a function of the Shannon entropy H(X), have been respectively derived by Massey [77] and by McEliece and Yu [78], followed by a derivation of improved upper and lower bounds by De Santis et al. [80]. More generally, given a probability mass function $P_{X}$ on X, it is of interest to minimize the generalized guessing moment $E\left[g^{\rho }\left(X\right)\right]=\sum_{x\in X}P_{X}\left(x\right)g^{\rho }\left(x\right)$ for ρ>0. For an arbitrary positive ρ, the ρ-th moment of the number of guesses is minimized by selecting the guessing function to be a ranking function $g_{X}$, for which $g_{X}\left(x\right)=\ell$ if $P_{X}\left(x\right)$ is the *ℓ*-th largest mass [77]. Although the tie breaking affects the choice of $g_{X}$, the distribution of $g_{X}\left(X\right)$ does not depend on how ties are resolved. Not only does this strategy minimize the average number of guesses, but it also minimizes the ρ-th moment of the number of guesses for every ρ>0. Upper and lower bounds on the ρ-th moment of ranking functions, expressed in terms of the Rényi entropies, were derived by Arikan [35], Boztaş [71], followed by recent improvements in the non-asymptotic regime by Sason and Verdú [56]. Although if |X| is small, it is straightforward to evaluate numerically the guessing moments, the benefit of bounds expressed in terms of Rényi entropies is particularly relevant when dealing with a random vector $X^{k}=\left(X_{1},\ldots ,X_{k}\right)$ whose letters belong to a finite alphabet X; computing all the probabilities of the mass function $P_{X^{k}}$ over the set $X^{k}$, and then sorting them in decreasing order for the calculation of the ρ-th moment of the optimal guessing function for the elements of $X^{k}$ becomes infeasible even for moderate values of *k*. In contrast, regardless of the value of *k*, bounds on guessing moments which depend on the Rényi entropy are readily computable if for example, ${\left\{X_{i}\right\}}_{i=1}^{k}$ are independent; in which case, the Rényi entropy of the vector is equal to the sum of the Rényi entropies of its components. Arikan’s bounds in [35] are asymptotically tight for random vectors of length *k* as k→∞, thus providing the correct exponential growth rate of the guessing moments for sufficiently large *k*.

#### 5.1.2. Analysis

We next analyze the following setup of guessing. Let ${\left\{X_{i}\right\}}_{i=1}^{k}$ be i.i.d. random variables where $X_{1}∼P_{X}$ takes values on a finite set X with |X|=n. To cluster the data [82] (see also [12] (Section 3.A) and references therein), suppose that each $X_{i}$ is mapped to $Y_{i}=f\left(X_{i}\right)$ where $f\in F_{n,m}$ is an arbitrary deterministic function (independent of the index *i*) with m<n. Consequently, ${\left\{Y_{i}\right\}}_{i=1}^{k}$ are i.i.d., and each $Y_{i}$ takes values on a finite set Y with |Y|=m<|X|.

Let $g_{X^{k}}:X^{k}\to \left\{1,\ldots ,n^{k}\right\}$ and $g_{Y^{k}}:Y^{k}\to \left\{1,\ldots ,m^{k}\right\}$ be, respectively, the ranking functions of the random vectors $X^{k}=\left(X_{1},\ldots ,X_{k}\right)$ and $Y^{k}=\left(Y_{1},\ldots ,Y_{k}\right)$ by sorting in separate decreasing orders the probabilities $P_{X^{k}}\left(x^{k}\right)=\prod_{i=1}^{k}P_{X}\left(x_{i}\right)$ for $x^{k}\in X^{k}$, and $P_{Y^{k}}\left(y^{k}\right)=\prod_{i=1}^{k}P_{Y}\left(y_{i}\right)$ for $y^{k}\in Y^{k}$ where ties in both cases are resolved arbitrarily. In view of Arikan’s bounds on the ρ-th moment of ranking functions (see [35] (Theorem 1) for the lower bound, and [35] (Proposition 4) for the upper bound), since $|X^{k}|=n^{k}$ and $|Y^{k}|=m^{k}$, the following bounds hold for all ρ>0:

(63) $$\begin{array}{c} \rho H_{\frac{1}{1+\rho }}\left(X\right)-\frac{\rho \log(1+k\ln n)}{k}\leq \frac{1}{k}\;\log E\left[g_{X^{k}}^{\rho }\left(X^{k}\right)\right]\leq \rho H_{\frac{1}{1+\rho }}\left(X\right), \end{array}$$

(64) $$\begin{array}{c} \rho H_{\frac{1}{1+\rho }}\left(Y\right)-\frac{\rho \log(1+k\ln m)}{k}\leq \frac{1}{k}\;\log E\left[g_{Y^{k}}^{\rho }\left(Y^{k}\right)\right]\leq \rho H_{\frac{1}{1+\rho }}\left(Y\right). \end{array}$$

In the following, we rely on Theorem 2 and the bounds in (63) and (64) to obtain bounds on the exponential reduction of the ρ-th moment of the ranking function of $X^{k}$ as a result of its mapping to $Y^{k}$. First, the combination of (63) and (64) yields

(65) $$\begin{array}{c} \rho \left[H_{\frac{1}{1+\rho }}\left(X\right)-H_{\frac{1}{1+\rho }}\left(Y\right)\right]-\frac{\rho \log(1+k\ln n)}{k}\\ \leq \frac{1}{k}\log\frac{E\left[g_{X^{k}}^{\rho }\left(X^{k}\right)\right]}{E\left[g_{Y^{k}}^{\rho }\left(Y^{k}\right)\right]} \end{array}$$

(66) $$\begin{array}{c} \leq \rho \left[H_{\frac{1}{1+\rho }}\left(X\right)-H_{\frac{1}{1+\rho }}\left(Y\right)\right]+\frac{\rho \log(1+k\ln m)}{k}. \end{array}$$

In view of Theorem 2-(a) and (65), it follows that for an arbitrary $f\in F_{n,m}$ and ρ>0

(67) $$\begin{array}{c} \frac{1}{k}\log\frac{E\left[g_{X^{k}}^{\rho }\left(X^{k}\right)\right]}{E\left[g_{Y^{k}}^{\rho }\left(Y^{k}\right)\right]}\geq \rho \left[H_{\frac{1}{1+\rho }}\left(X\right)-H_{\frac{1}{1+\rho }}\left({\tilde{X}}_{m}\right)\right]-\frac{\rho \log(1+k\ln n)}{k} \end{array}$$

where ${\tilde{X}}_{m}$ is a random variable whose probability mass function is given in (30). Please note that

(68) $$\begin{array}{c} H_{\frac{1}{1+\rho }}\left({\tilde{X}}_{m}\right)\leq H_{\frac{1}{1+\rho }}\left(X\right),\;\frac{\rho \log(1+k\ln n)}{k}\underset{k\to \infty }{⟶}0 \end{array}$$

where the first inequality in (68) holds since $P_{X}≺P_{{\tilde{X}}_{m}}$ (see Lemma 5) and the Rényi entropy is Schur-concave.

By the explicit construction of the function $f^{*}\in F_{n,m}$ according to the algorithm in Steps 1–4 in the proof of Theorem 2 (based on the Huffman procedure), by setting $Y_{i}:=f^{*}\left(X_{i}\right)$ for every i∈{1,…,k}, it follows from (34) and (66) that for all ρ>0

(69) $$\begin{array}{c} \frac{1}{k}\log\frac{E\left[g_{X^{k}}^{\rho }\left(X^{k}\right)\right]}{E\left[g_{Y^{k}}^{\rho }\left(Y^{k}\right)\right]}\leq \rho \left[H_{\frac{1}{1+\rho }}\left(X\right)-H_{\frac{1}{1+\rho }}\left({\tilde{X}}_{m}\right)+v\;\left(\frac{1}{1+\rho }\right)\right]+\frac{\rho \log(1+k\ln m)}{k} \end{array}$$

where the monotonically increasing function v:(0,∞)→(0,∞) is given in (33), and it is depicted by the solid line in the left plot of Figure 2. In view of (33), it can be shown that the linear approximation v(α)≈v(1)α is excellent for all α∈[0,1], and therefore for all ρ>0

(70) $$\begin{array}{c} v\;\left(\frac{1}{1+\rho }\right)\approx \frac{0.08607}{1+\rho }\;\mathrm{bits}. \end{array}$$

Hence, for sufficiently large value of *k*, the gap between the lower and upper bounds in (67) and (69) is marginal, being approximately equal to $\frac{0.08607\;\rho }{1+\rho }\;\mathrm{bits}$ for all ρ>0.

The following theorem summarizes our result in this section.

**Theorem** **3.** *Let*

- *${\left\{X_{i}\right\}}_{i=1}^{k}$ be i.i.d. with $X_{1}∼P_{X}$ taking values on a set X with |X|=n;*
- *$Y_{i}=f\left(X_{i}\right)$, for every i∈{1,…,k}, where $f\in F_{n,m}$ is a deterministic function with m<n;*
- *$g_{X^{k}}:X^{k}\to \left\{1,\ldots ,n^{k}\right\}$ and $g_{Y^{k}}:Y^{k}\to \left\{1,\ldots ,m^{k}\right\}$ be, respectively, ranking functions of the random vectors $X^{k}=\left(X_{1},\ldots ,X_{k}\right)$ and $Y^{k}=\left(Y_{1},\ldots ,Y_{k}\right)$.*

*Then, for every ρ>0,*

*(a)*  *The lower bound in (67) holds for every deterministic function $f\in F_{n,m}$;*
*(b)*  *The upper bound in (69) holds for the specific $f^{*}\in F_{n,m}$, whose construction relies on the Huffman algorithm (see Steps 1–4 of the procedure in the proof of Theorem 2);*
*(c)*  *The gap between these bounds, for $f=f^{*}$ and sufficiently large k, is at most $\rho \;v\;\left(\frac{1}{1+\rho }\right)\approx \frac{0.08607\;\rho }{1+\rho }\;bits.$*

#### 5.1.3. Numerical Result

The following simple example illustrates the tightness of the achievable upper bound and the universal lower bound in Theorem 3, especially for sufficiently long sequences.

**Example** **1.** *Let X be geometrically distributed restricted to {1,…,n} with the probability mass function*

(71) $$\begin{array}{c} P_{X}\left(j\right)=\frac{\left(1-a\right)\;a^{j-1}}{1-a^{n}},\;j\in \left\{1,\ldots ,n\right\} \end{array}$$

**Figure 4:**
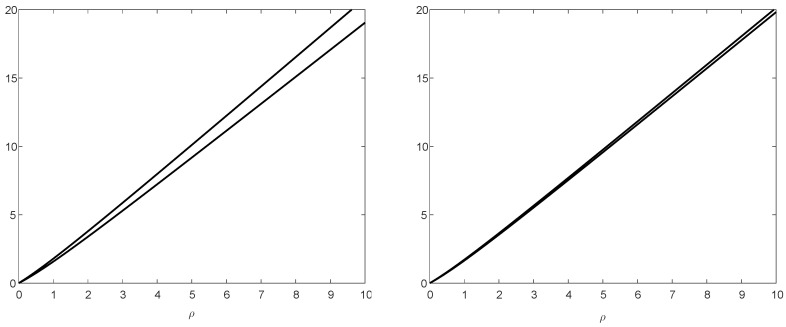
Plots of the upper and lower bounds on $\frac{1}{k}\log_{2}\frac{E\left[g_{X^{k}}^{\rho }\left(X^{k}\right)\right]}{E\left[g_{Y^{k}}^{\rho }\left(Y^{k}\right)\right]}$ in Theorem 3, as a function of ρ>0, for random vectors of length k=100 (left plot) or k=1000 (right plot) in the setting of Example 1. Each plot shows the universal lower bound for an arbitrary deterministic $f\in F_{128,\;16}$, and the achievable upper bound with the construction of the deterministic function $f=f^{*}\in F_{128,\;16}$ (based on the Huffman algorithm) in Theorem 3 (see, respectively, (67) and (69)).

*where $a=\frac{24}{25}$ and n=128. Assume that $X_{1},\ldots ,X_{k}$ are i.i.d. with $X_{1}∼P_{X}$, and let $Y_{i}=f\left(X_{i}\right)$ for a deterministic function $f\in F_{n,m}$ with n=128 and m=16. We compare the upper and lower bounds in Theorem 3 for the two cases where the sequence $X^{k}=\left(X_{1},\ldots ,X_{k}\right)$ is of length k=100 or k=1000. The lower bound in (67) holds for an arbitrary deterministic $f\in F_{n,m}$, and the achievable upper bound in (69) holds for the construction of the deterministic function $f=f^{*}\in F_{n,m}$ (based on the Huffman algorithm) in Theorem 3. Numerical results are shown in Figure 4, providing plots of the upper and lower bounds on $\frac{1}{k}\log_{2}\frac{E\left[g_{X^{k}}^{\rho }\left(X^{k}\right)\right]}{E\left[g_{Y^{k}}^{\rho }\left(Y^{k}\right)\right]}$ in Theorem 3, and illustrating the improved tightness of these bounds when the value of k is increased from 100 (left plot) to 1000 (right plot). From Theorem 3-(c), for sufficiently large k, the gap between the upper and lower bounds is less than 0.08607 bits (for all ρ>0); this is consistent with the right plot of Figure 4 where k=1000.*

### 5.2. Lossless Source Coding

#### 5.2.1. Background

For uniquely decodable (UD) lossless source coding, Campbell [51,83] proposed the cumulant generating function of the codeword lengths as a generalization to the frequently used design criterion of average code length. Campbell’s motivation in [51] was to control the contribution of the longer codewords via a free parameter in the cumulant generating function: if the value of this parameter tends to zero, then the resulting design criterion becomes the average code length per source symbol; on the other hand, by increasing the value of the free parameter, the penalty for longer codewords is more severe, and the resulting code optimization yields a reduction in the fluctuations of the codeword lengths.

We introduce the coding theorem by Campbell [51] for lossless compression of a discrete memoryless source (DMS) with UD codes, which serves for our analysis jointly with Theorem 2.

**Theorem** **4**  (Campbell 1965, [51])**.** *Consider a DMS which emits symbols with a probability mass function $P_{X}$ defined on a (finite or countably infinite) set X. Consider a UD fixed-to-variable source code operating on source sequences of k symbols with an alphabet of the codewords of size D. Let $\ell (x^{k})$ be the length of the codeword which corresponds to the source sequence $x^{k}:=\left(x_{1},\ldots ,x_{k}\right)\in X^{k}$. Consider the scaled cumulant generating function of the codeword lengths*

(72) $$\begin{array}{c} \Lambda_{k}\left(\rho \right):=\frac{1}{k}\;\log_{D}\left(\;\sum_{x^{k}\in X^{k}}P_{X^{k}}\left(x^{k}\right)\;D^{\rho \;\ell (x^{k})}\right),\;\rho >0 \end{array}$$

*where*

(73) $$\begin{array}{c} P_{X^{k}}\left(x^{k}\right)=\prod_{i=1}^{k}P_{X}\left(x_{i}\right),\;\forall \;x^{k}\in X^{k}. \end{array}$$

*Then, for every ρ>0, the following hold:*

*(a)*  *Converse result:*
  (74) $$\begin{array}{c} \frac{\Lambda_{k}\left(\rho \right)}{\rho }\geq \frac{1}{\log D}\;H_{\frac{1}{1+\rho }}\left(X\right). \end{array}$$
*(b)*  *Achievability result: there exists a UD source code, for which*
  (75) $$\begin{array}{c} \frac{\Lambda_{k}\left(\rho \right)}{\rho }\leq \frac{1}{\log D}\;H_{\frac{1}{1+\rho }}\left(X\right)+\frac{1}{k}. \end{array}$$

The term scaled cumulant generating function is used in view of [56] (Remark 20). The bounds in Theorem 4, expressed in terms of the Rényi entropy, imply that for sufficiently long source sequences, it is possible to make the scaled cumulant generating function of the codeword lengths approach the Rényi entropy as closely as desired by a proper fixed-to-variable UD source code; moreover, the converse result shows that there is no UD source code for which the scaled cumulant generating function of its codeword lengths lies below the Rényi entropy. By invoking L’Hôpital’s rule, one gets from (72)

(76) $$\begin{array}{c} \lim_{\rho ↓0}\frac{\Lambda_{k}\left(\rho \right)}{\rho }=\frac{1}{k}\sum_{x^{k}\in X^{k}}P_{X^{k}}\left(x^{k}\right)\;\ell \left(x^{k}\right)=\frac{1}{k}\;E\left[\ell \left(X^{k}\right)\right]. \end{array}$$

Hence, by letting ρ tend to zero in (74) and (75), it follows from (4) that Campbell’s result in Theorem 4 generalizes the well-known bounds on the optimal average length of UD fixed-to-variable source codes (see, e.g., [84] ((5.33) and (5.37))):

(77) $$\begin{array}{c} \frac{1}{\log D}\;H\left(X\right)\leq \frac{1}{k}\;E\left[\ell \left(X^{k}\right)\right]\leq \frac{1}{\log D}\;H\left(X\right)+\frac{1}{k}, \end{array}$$

and (77) is satisfied by Huffman coding (see, e.g., [84] (Theorem 5.8.1)). Campbell’s result therefore generalizes Shannon’s fundamental result in [85] for the average codeword lengths of lossless compression codes, expressed in terms of the Shannon entropy.

Following the work by Campbell [51], Courtade and Verdú derived in [52] non-asymptotic bounds for the scaled cumulant generating function of the codeword lengths for $P_{X}$-optimal variable-length lossless codes [23,86]. These bounds were used in [52] to obtain simple proofs of the asymptotic normality of the distribution of codeword lengths, and the reliability function of memoryless sources allowing countably infinite alphabets. Sason and Verdú recently derived in [56] improved non-asymptotic bounds on the cumulant generating function of the codeword lengths for fixed-to-variable optimal lossless source coding without prefix constraints, and non-asymptotic bounds on the reliability function of a DMS, tightening the bounds in [52].

#### 5.2.2. Analysis

The following analysis for lossless source compression with UD codes relies on a combination of Theorems 2 and 4.

Let $X_{1},\ldots ,X_{k}$ be i.i.d. symbols which are emitted from a DMS according to a probability mass function $P_{X}$ whose support is a finite set X with |X|=n. Similarly to Section 5.1, to cluster the data, suppose that each symbol $X_{i}$ is mapped to $Y_{i}=f\left(X_{i}\right)$ where $f\in F_{n,m}$ is an arbitrary deterministic function (independent of the index *i*) with m<n. Consequently, the i.i.d. symbols $Y_{1},\ldots ,Y_{k}$ take values on a set Y with |Y|=m<|X|. Consider two UD fixed-to-variable source codes: one operating on the sequences $x^{k}\in X^{k}$, and the other one operates on the sequences $y^{k}\in Y^{k}$; let *D* be the size of the alphabets of both source codes. Let $\ell (x^{k})$ and $\bar{\ell }\left(y^{k}\right)$ denote the length of the codewords for the source sequences $x^{k}$ and $y^{k}$, respectively, and let $\Lambda_{k}\left(\cdot \right)$ and ${\bar{\Lambda }}_{k}\left(\cdot \right)$ denote their corresponding scaled cumulant generating functions (see (72)).

In view of Theorem 4-(b), for every ρ>0, there exists a UD source code for the sequences in $X^{k}$ such that the scaled cumulant generating function of its codeword lengths satisfies (75). Furthermore, from Theorem 4-(a), we get

(78) $$\begin{array}{c} \frac{{\bar{\Lambda }}_{k}\left(\rho \right)}{\rho }\geq \frac{1}{\log D}\;H_{\frac{1}{1+\rho }}\left(Y\right). \end{array}$$

From (75), (78) and Theorem 2 (a) and (b), for every ρ>0, there exist a UD source code for the sequences in $X^{k}$, and a construction of a deterministic function $f\in F_{n,m}$ (as specified by Steps 1–4 in the proof of Theorem 2, borrowed from [12]) such that the difference between the two scaled cumulant generating functions satisfies

(79) $$\begin{array}{c} \Lambda_{k}\left(\rho \right)-{\bar{\Lambda }}_{k}\left(\rho \right)\leq \frac{\rho }{\log D}\left[H_{\frac{1}{1+\rho }}\left(X\right)-H_{\frac{1}{1+\rho }}\left({\tilde{X}}_{m}\right)+v\;\left(\frac{1}{1+\rho }\right)\right]+\frac{\rho }{k}, \end{array}$$

where (79) holds for every UD source code operating on the sequences in $Y^{k}$ with $Y_{i}=f\left(X_{i}\right)$ (for i=1,…,k) and the specific construction of $f\in F_{n,m}$ as above, and ${\tilde{X}}_{m}$ in the right side of (79) is a random variable whose probability mass function is given in (30). The right side of (79) can be very well approximated (for all ρ>0) by using (70).

We proceed with a derivation of a lower bound on the left side of (79). In view of Theorem 4, it follows that (74) is satisfied for every UD source code which operates on the sequences in $X^{k}$; furthermore, Theorems 2 and 4 imply that, for every $f\in F_{n,m}$, there exists a UD source code which operates on the sequences in $Y^{k}$ such that

(80) $$\begin{array}{cc} \frac{{\bar{\Lambda }}_{k}\left(\rho \right)}{\rho } & \leq \frac{1}{\log D}\;H_{\frac{1}{1+\rho }}\left(Y\right)+\frac{1}{k}, \end{array}$$

(81) $$\begin{array}{cc} & \leq \frac{1}{\log D}\;H_{\frac{1}{1+\rho }}\left({\tilde{X}}_{m}\right)+\frac{1}{k}, \end{array}$$

where (81) is due to (39) since $Y_{i}=f\left(X_{i}\right)$ (for i=1,…,k) with an arbitrary deterministic function $f\in F_{n,m}$, and $Y_{i}∼P_{Y}$ for every *i*; hence, from (74), (80) and (81),

(82) $$\begin{array}{c} \Lambda_{k}\left(\rho \right)-{\bar{\Lambda }}_{k}\left(\rho \right)\geq \frac{\rho }{\log D}\left(H_{\frac{1}{1+\rho }}\left(X\right)-H_{\frac{1}{1+\rho }}\left({\tilde{X}}_{m}\right)\right)-\frac{\rho }{k}. \end{array}$$

We summarize our result as follows.

**Theorem** **5.** *Let*

- *$X_{1},\ldots ,X_{k}$ be i.i.d. symbols which are emitted from a DMS according to a probability mass function $P_{X}$ whose support is a finite set X with |X|=n;*
- *Each symbol $X_{i}$ be mapped to $Y_{i}=f\left(X_{i}\right)$ where $f\in F_{n,m}$ is the deterministic function (independent of the index i) with m<n, as specified by Steps 1–4 in the proof of Theorem 2 (borrowed from [12]);*
- *Two UD fixed-to-variable source codes be used: one code encodes the sequences $x^{k}\in X^{k}$, and the other code encodes their mappings $y^{k}\in Y^{k}$; let the common size of the alphabets of both codes be D;*
- *$\Lambda_{k}\left(\cdot \right)$ and ${\bar{\Lambda }}_{k}\left(\cdot \right)$ be, respectively, the scaled cumulant generating functions of the codeword lengths of the k-length sequences in $X^{k}$ (see (72)) and their mapping to $Y^{k}$.*

*Then, for every ρ>0, the following holds for the difference between the scaled cumulant generating functions $\Lambda_{k}\left(\cdot \right)$ and ${\bar{\Lambda }}_{k}\left(\cdot \right)$:*

*(a)*  *There exists a UD source code for the sequences in $X^{k}$ such that the upper bound in (79) is satisfied for every UD source code which operates on the sequences in $Y^{k}$;*
*(b)*  *There exists a UD source code for the sequences in $Y^{k}$ such that the lower bound in (82) holds for every UD source code for the sequences in $X^{k}$; furthermore, the lower bound in (82) holds in general for every deterministic function $f\in F_{n,m}$;*
*(c)*  *The gap between the upper and lower bounds in (79) and (82), respectively, is at most $\frac{\rho }{\log D}\;v\;\left(\frac{1}{1+\rho }\right)+\frac{2\rho }{k}$ (the function v:(0,∞)→(0,∞) is introduced in (33)), which is approximately $\frac{0.08607\rho \;\log_{D}2}{1+\rho }+\frac{2\rho }{k}$;*
*(d)*  *The UD source codes in Items (a) and (b) for the sequences in $X^{k}$ and $Y^{k}$, respectively, can be constructed to be prefix codes by the algorithm in Remark 11.*

**Remark** **11** (An Algorithm for Theorem 5 (d))**.** *A construction of the UD source codes for the sequences in $X^{k}$ and $Y^{k}$, whose existence is assured by Theorem 5 (a) and (b) respectively, is obtained by the following algorithm (of three steps) which also constructs them as prefix codes:*

*(1)*  *As a preparatory step, we first calculate the probability mass function $P_{Y}$ from the given probability mass function $P_{X}$ and the deterministic function $f\in F_{n,m}$ which is obtained by Steps 1–4 in the proof of Theorem 2; accordingly, $P_{Y}\left(y\right)=\sum_{x\in X:\;f(x)=y}P_{X}\left(x\right)$ for all y∈Y. We then further calculate the probability mass functions for the i.i.d. sequences in $X^{k}$ and $Y^{k}$ (see (73)); recall that the number of types in $X^{k}$ and $Y^{k}$ is polynomial in k (being upper bounded by ${\left(k+1\right)}^{n-1}$ and ${\left(k+1\right)}^{m-1}$, respectively), and the values of these probability mass functions are fixed over each type;*
*(2)*  *The sets of codeword lengths of the two UD source codes, for the sequences in $X^{k}$ and $Y^{k}$, can (separately) be designed according to the achievability proof in Campbell’s paper (see [51] (p. 428)). More explicitly, let $\alpha :=\frac{1}{1+\rho }$; for all $x^{k}\in X^{k}$, let $\ell (x^{k})\in N$ be given by*
  (83) $$\begin{array}{c} \ell \left(x^{k}\right)=\left\lceil -\alpha \log_{D}P_{X^{k}}\left(x^{k}\right)+\log_{D}Q_{k}\right\rceil \end{array}$$
  *with*
  (84) $$\begin{array}{c} Q_{k}:=\sum_{x^{k}\in X^{k}}P_{X^{k}}^{\alpha }\left(x^{k}\right)={\left(\sum_{x\in X}P_{X}^{\alpha }\left(x\right)\right)}^{k}, \end{array}$$
  *and let $\bar{\ell }\left(y^{k}\right)\in N$, for all $y^{k}\in Y^{k}$, be given similarly to (83) and (84) by replacing $P_{X}$ with $P_{Y}$, and $P_{X^{k}}$ with $P_{Y^{k}}$. This suggests codeword lengths for the two codes which fulfil (75) and (80), and also, both satisfy Kraft’s inequality;*
*(3)*  *The separate construction of two prefix codes (a.k.a. instantaneous codes) based on their given sets of codeword lengths ${\left\{\ell \left(x^{k}\right)\right\}}_{x^{k}\in X^{k}}$ and ${\left\{\bar{\ell }\left(y^{k}\right)\right\}}_{y^{k}\in Y^{k}}$, as determined in Step 2, is standard (see, e.g., the construction in the proof of [84] (Theorem 5.2.1)).*

Theorem 5 is of interest since it provides upper and lower bounds on the reduction in the cumulant generating function of close-to-optimal UD source codes because of clustering data, and Remark 11 suggests an algorithm to construct such UD codes which are also prefix codes. For long enough sequences (as k→∞), the upper and lower bounds on the difference between the scaled cumulant generating functions of the suggested source codes for the original and clustered data almost match (see (79) and (82)), being roughly equal to $\rho \left(H_{\frac{1}{1+\rho }}\left(X\right)-H_{\frac{1}{1+\rho }}\left({\tilde{X}}_{m}\right)\right)$ (with logarithms on base *D*, which is the alphabet size of the source codes); as k→∞, the gap between these upper and lower bounds is less than $0.08607\log_{D}2$. Furthermore, in view of (76),

(85) $$\begin{array}{c} \lim_{\rho ↓0}\frac{\Lambda_{k}\left(\rho \right)-{\bar{\Lambda }}_{k}\left(\rho \right)}{\rho }=\frac{1}{k}\left(E\left[\ell \left(X^{k}\right)\right]-E\left[\bar{\ell }\left(Y^{k}\right)\right]\right), \end{array}$$

so, it follows from (4), (33), (79) and (82) that the difference between the average code lengths (normalized by *k*) of the original and clustered data satisfies

(86) $$\begin{array}{c} \frac{H\left(X\right)-H\left({\tilde{X}}_{m}\right)}{\log D}-\frac{1}{k}\leq \frac{E\left[\ell \left(X^{k}\right)\right]-E\left[\bar{\ell }\left(Y^{k}\right)\right]}{k}\leq \frac{H\left(X\right)-H\left({\tilde{X}}_{m}\right)+0.08607\log2}{\log D}, \end{array}$$

where the gap between the upper and lower bounds is equal to $0.08607\log_{D}2+\frac{1}{k}$.

## Appendix A. Proof of Lemma 2

We first find the extreme values of $p_{\min}$ under the assumption that $P\in P_{n}\left(\rho \right)$. If $\frac{p_{\max}}{p_{\min}}=1$, then *P* is the equiprobable distribution on X and $p_{\min}=\frac{1}{n}$. On the other hand, if $\frac{p_{\max}}{p_{\min}}=\rho$, then the minimal possible value of $p_{\min}$ is obtained when *P* is the one-odd-mass distribution with n−1 masses equal to $\rho p_{\min}$ and a smaller mass equal to $p_{\min}$. The latter case yields $p_{\min}=\frac{1}{1+(n-1)\rho }$.

Let $\beta :=p_{\min}$, so β can get any value in the interval $\left[\frac{1}{1+(n-1)\rho },\;\frac{1}{n}\right]:=\Gamma_{\rho }^{\left(n\right)}$. From Lemma 1, $P≺Q_{\beta }$ and $Q_{\beta }\in P_{n}\left(\rho \right)$, and the Schur-concavity of the Rényi entropy yields $H_{\alpha }\left(P\right)\geq H_{\alpha }\left(Q_{\beta }\right)$ for all $P\in P_{n}\left(\rho \right)$ with $p_{\min}=\beta$. Minimizing $H_{\alpha }\left(P\right)$ over $P\in P_{n}\left(\rho \right)$ can be therefore restricted to minimizing $H_{\alpha }\left(Q_{\beta }\right)$ over $\beta \in \Gamma_{\rho }^{\left(n\right)}$.

## Appendix B. Proof of Lemma 3

The sequence ${\left\{c_{\alpha }^{\left(n\right)}\left(\rho \right)\right\}}_{n\in N}$ is non-negative since $H_{\alpha }\left(P\right)\leq \log n$ for all $P\in P_{n}$. To prove (17),

(A1) $$\begin{array}{cc} 0\leq c_{\alpha }^{\left(n\right)}\left(\rho \right) & =\log n-\min_{P\in P_{n}\left(\rho \right)}H_{\alpha }\left(P\right) \end{array}$$

(A2) $$\begin{array}{cc} & \leq \log n-\min_{P\in P_{n}\left(\rho \right)}H_{\infty }\left(P\right) \end{array}$$

(A3) $$\begin{array}{cc} & \leq \log n-\log\frac{n}{\rho }=\log\rho \end{array}$$

where (A2) holds since $H_{\alpha }\left(P\right)$ is monotonically decreasing in α, and (A3) is due to (5) and $p_{\max}\leq \frac{\rho }{n}$.

Let $U_{n}$ denote the equiprobable probability mass function on {1,…,n}. By the identity

(A4) $$\begin{array}{c} D_{\alpha }\left(P\|U_{n}\right)=\log n-H_{\alpha }\left(P\right), \end{array}$$

and since, by Lemma 2, $H_{\alpha }\left(\cdot \right)$ attains its minimum over the set of probability mass functions $P_{n}\left(\rho \right)$, it follows that $D_{\alpha }\left(\cdot \|U_{n}\right)$ attains its maximum over this set. Let $P^{*}\in P_{n}\left(\rho \right)$ be the probability measure which achieves the minimum in $c_{\alpha }^{\left(n\right)}\left(\rho \right)$ (see (16)), then from (A4)

(A5) $$\begin{array}{cc} c_{\alpha }^{\left(n\right)}\left(\rho \right) & =\max_{P\in P_{n}\left(\rho \right)}D_{\alpha }\left(P\|U_{n}\right) \end{array}$$

(A6) $$\begin{array}{c} =D_{\alpha }\left(P^{*}\|U_{n}\right). \end{array}$$

Let $Q^{*}$ be the probability mass function which is defined on {1,…,2n} as follows:

(A7) $$Q^{*}\left(i\right)=\{\begin{array}{cc} \frac{1}{2}\;P^{*}\left(i\right), & i\in \{1,\ldots ,n\},\\ \frac{1}{2}\;P^{*}\left(i-n\right), & i\in \{n+1,\ldots ,2n\}. \end{array}$$

Since by assumption $P^{*}\in P_{n}\left(\rho \right)$, it is easy to verify from (A7) that

(A8) $$\begin{array}{c} Q^{*}\in P_{2n}\left(\rho \right). \end{array}$$

Furthermore, from (A7),

(A9) $$\begin{array}{cc} D_{\alpha }\left(Q^{*}\|U_{2n}\right) & =\frac{1}{\alpha -1}\;\log\left(\sum_{i=1}^{2n}{\left(Q^{*}\left(i\right)\right)}^{\alpha }{\left(\frac{1}{2n}\right)}^{1-\alpha }\right) \end{array}$$

(A10) $$\begin{array}{cc} & =\frac{1}{\alpha -1}\log\left(\frac{1}{2}\;\sum_{i=1}^{n}{\left(P^{*}\left(i\right)\right)}^{\alpha }{\left(\frac{1}{n}\right)}^{1-\alpha }+\frac{1}{2}\sum_{i=n+1}^{2n}{\left(P^{*}\left(i-n\right)\right)}^{\alpha }{\left(\frac{1}{n}\right)}^{1-\alpha }\right) \end{array}$$

(A11) $$\begin{array}{cc} & =\frac{1}{\alpha -1}\log\left(\sum_{i=1}^{n}{\left(P^{*}\left(i\right)\right)}^{\alpha }{\left(\frac{1}{n}\right)}^{1-\alpha }\right) \end{array}$$

(A12) $$\begin{array}{cc} & =D_{\alpha }\left(P^{*}\|U_{n}\right). \end{array}$$

Combining (A5)–(A12) yields

(A13) $$\begin{array}{cc} c_{\alpha }^{\left(2n\right)}\left(\rho \right) & =\max_{Q\in P_{2n}\left(\rho \right)}D_{\alpha }\left(Q\|U_{2n}\right) \end{array}$$

(A14) $$\begin{array}{cc} & \geq D_{\alpha }\left(Q^{*}\|U_{2n}\right) \end{array}$$

(A15) $$\begin{array}{cc} & =D_{\alpha }\left(P^{*}\|U_{n}\right) \end{array}$$

(A16) $$\begin{array}{cc} & =c_{\alpha }^{\left(n\right)}\left(\rho \right), \end{array}$$

proving (18). Finally, in view of (A5), $c_{\alpha }^{\left(n\right)}\left(\rho \right)$ is monotonically increasing in α since so is the Rényi divergence of order α (see [87] (Theorem 3)).

## Appendix C. Proof of Lemma 4

From Lemma 2, the minimizing distribution of $H_{\alpha }$ is given by $Q_{\beta }\in P_{n}\left(\rho \right)$ where

(A17) $$\begin{array}{c} Q_{\beta }=\left(\underset{i}{\underset{︸}{\;\rho \beta ,\ldots ,\rho \beta }},\;1-\left(n+i\rho -i-1\right)\beta ,\;\underset{n-i-1}{\underset{︸}{\beta ,\beta ,\ldots ,\beta }}\right) \end{array}$$

with $\beta \in \left[\frac{1}{1+(n-1)\rho },\frac{1}{n}\right]$, and $1-\left(n+i\rho -i-1\right)\beta \leq \rho \beta \leq \frac{\rho }{n}$. It therefore follows that the influence of the middle probability mass of $Q_{\beta }$ on $H_{\alpha }\left(Q_{\beta }\right)$ tends to zero as n→∞. Therefore, in this asymptotic case, one can instead minimize $H_{\alpha }\left({\tilde{Q}}_{m}\right)$ where

(A18) $$\begin{array}{c} {\tilde{Q}}_{m}=\left(\underset{m}{\underset{︸}{\;\rho \beta ,\ldots ,\rho \beta }},\;\underset{n-m}{\underset{︸}{\beta ,\beta ,\ldots ,\beta }}\right) \end{array}$$

with the free parameter m∈{1,…,n} and $\beta =\frac{1}{n+m(\rho -1)}$ (so that the total mass of ${\tilde{Q}}_{m}$ is equal to 1).

For α∈(0,1)∪(1,∞), straightforward calculation shows that

(A19) $$\begin{array}{cc} H_{\alpha }\left({\tilde{Q}}_{m}\right) & =\frac{1}{1-\alpha }\;\log\left(\sum_{j=1}^{n}{\tilde{Q}}_{m}^{\alpha }\left(j\right)\right)\\ & =\log n-\frac{1}{\alpha -1}\log\left(\frac{1+\frac{m}{n}\;\left(\rho^{\alpha }-1\right)}{{\left(1+\frac{m}{n}\;\left(\rho -1\right)\right)}^{\alpha }}\right), \end{array}$$

and by letting n→∞, the limit of the sequence ${\left\{c_{\alpha }^{\left(n\right)}\left(\rho \right)\right\}}_{n\in N}$ exists, and it is equal to

(A20) $$\begin{array}{cc} c_{\alpha }^{\left(\infty \right)}\left(\rho \right) & :=\lim_{n\to \infty }c_{\alpha }^{\left(n\right)}\left(\rho \right)\\ & =\lim_{n\to \infty }\left(\log n-\min_{m\in \{1,\ldots ,n\}}H_{\alpha }\left({\tilde{Q}}_{m}\right)\right)\\ & =\lim_{n\to \infty }\max_{m\in \{1,\ldots ,n\}}\left\{\frac{1}{\alpha -1}\;\log\left(\frac{1+\frac{m}{n}\;\left(\rho^{\alpha }-1\right)}{{\left(1+\frac{m}{n}\;\left(\rho -1\right)\right)}^{\alpha }}\right)\right\}\\ & =\max_{x\in [0,1]}\left\{\frac{1}{\alpha -1}\;\log\left(\frac{1+(\rho^{\alpha }-1)x}{{\left(1+(\rho -1)x\right)}^{\alpha }}\right)\right\}. \end{array}$$

Let $f_{\alpha }:\left[0,1\right]\to R$ be given by

(A21) $$\begin{array}{c} f_{\alpha }\left(x\right)=\frac{1+(\rho^{\alpha }-1)x}{{\left(1+(\rho -1)x\right)}^{\alpha }},\;x\in \left[0,1\right]. \end{array}$$

Then, $f_{\alpha }\left(0\right)=f_{\alpha }\left(1\right)=1$, and straightforward calculation shows that the derivative $f_{\alpha }^{\prime }$ vanishes if and only if

(A22) $$\begin{array}{c} x=x^{*}:=\frac{1+\alpha \left(\rho -1\right)-\rho^{\alpha }}{\left(1-\alpha \right)\left(\rho -1\right)\left(\rho^{\alpha }-1\right)}. \end{array}$$

We rely here on a specialized version of the mean value theorem, known as Rolle’s theorem, which states that any real-valued differentiable function that attains equal values at two distinct points must have a point somewhere between them where the first derivative at this point is zero. By Rolle’s theorem, and due to the uniqueness of the point $x^{*}$ in (A22), it follows that $x^{*}\in \left(0,1\right)$. Substituting (A22) into (A20) gives (20). Taking the limit of (20) when α→∞ gives the result in (21).

In the limit where α→1, the Rényi entropy of order α tends to the Shannon entropy. Hence, letting α→1 in (20), it follows that for the Shannon entropy

(A23) $$\begin{array}{cc} c_{1}^{\left(\infty \right)}\left(\rho \right) & =\lim_{\alpha \to 1}c_{\alpha }^{\left(\infty \right)}\left(\rho \right)\\ & =\lim_{\alpha \to 1}\left\{\frac{1}{\alpha -1}\;\log\left(1+\frac{1+\alpha \;\left(\rho -1\right)-\rho^{\alpha }}{\left(1-\alpha )(\rho -1\right)}\right)-\frac{\alpha }{\alpha -1}\;\log\left(1+\frac{1+\alpha \;\left(\rho -1\right)-\rho^{\alpha }}{\left(1-\alpha \right)\left(\rho^{\alpha }-1\right)}\right)\right\}\\ & =\frac{\rho \log\rho }{\rho -1}-\log e-\log\left(\frac{\rho \log_{e}\rho }{\rho -1}\right), \end{array}$$

where (A23) follows by invoking L’Hôpital’s rule. This proves (22).

From (17)–(19), we get $0\leq c_{\alpha }^{\left(n\right)}\left(\rho \right)\leq c_{\alpha }^{\left(\infty \right)}\left(\rho \right)$. Since $c_{\alpha }^{\left(n\right)}\left(\rho \right)$ is monotonically increasing in α∈[0,∞], for every n∈N, so is $c_{\alpha }^{\left(\infty \right)}\left(\rho \right)$; hence, (21) yields $c_{\alpha }^{\left(\infty \right)}\left(\rho \right)\leq \log\rho$. This proves (24).
